# Performance of cervical cancer screening and triage strategies among women living with HIV in China

**DOI:** 10.1002/cam4.4152

**Published:** 2021-08-02

**Authors:** Rufei Duan, Xuelian Zhao, Hongyun Zhang, Xiaoqian Xu, Liuye Huang, Aihui Wu, Le Li, Youlin Qiao, Fanghui Zhao

**Affiliations:** ^1^ Department of Cancer Epidemiology National Cancer Center/National Clinical Research Center for Cancer/Cancer Hospital Chinese Academy of Medical Sciences and Peking Union Medical College Beijing China; ^2^ Department of Gynaecology and Obstetrics The First Affiliated Hospital of Kunming Medical University Kunming China; ^3^ Department of Gynaecology and Obstetrics The Third People's Hospital of Kunming Kunming China

**Keywords:** cervical cancer, HIV, performance, screening, triage

## Abstract

**Objectives:**

To evaluate the clinical performance of liquid‐based cytology (LBC), HPV tests and visual inspections with acetic acid or Lugol's iodine (VIA/VILI) as primary screening and triage strategies among Chinese women living with HIV (WLHIV).

**Methods:**

WLHIV aged 18 years and older were recruited from HIV/AIDS treatment clinic in Yunnan, China from 2019 to 2020. Women were screened with self‐ and physician‐sampling for HPV tests, LBC, and VIA/VILI. Women positive for any HPV or with cytological abnormalities were recalled for colposcopy examination and biopsy when necessary. Clinical performance of primary and triage strategies for detecting cervical intraepithelial neoplasia grade 2 or worse (CIN2+) was evaluated.

**Results:**

For primary screening, sensitivity of physician‐HPV tests was 100%, 89.5%, and 100% for hybrid capture 2 (HC2), cobas, and Sansure HPV, and specificity was 80.4%, 85.1%, and 72.0%, respectively. Self‐HPV test achieved considerable performance with physician‐HPV. Sensitivity and specificity were 61.1% and 96.3% for LBC (atypical squamous cells of undetermined significance or worse [ASCUS+]), 40.0% and 77.3% for VIA/VILI. For triaging HPV‐positive women, LBC (ASCUS+), HPV‐16/18 genotyping, and VIA/VILI‐elevated specificity with sensitivity declined 30%–50% compared with HPV screening alone. Restricted HPV genotyping triage (HPV‐16/18/31/33/45/52/58) demonstrated the optimal accuracy (89.5% sensitivity, 81.9% specificity), and was similar to HPV‐16/18 with reflex LBC (ASCUS+). Combination antiretroviral therapies (cARTs) <2 years were associated with decreased specificity of HC2 (aOR: 1.87, 95% CI: 1.22–3.91) and Sansure HPV (2.48, 1.43–4.29).

**Conclusions:**

Self‐HPV with restricted genotyping triage is highly recommended for cervical cancer screening for WLHIV in China. Feasible triage to increase HPV specificity among women with short duration of cART is needed.

## 1 INTRODUCTION

With the rollout of combination antiretroviral therapies (cARTs), the incidence of opportunistic infections, Kaposi's sarcoma and non‐Hodgkin's lymphoma have decreased in people infected with human immunodeficiency virus (HIV).^1^ However, the incidence of cervical cancer remains high among women living with HIV (WLHIV).^2^ WLHIV bear a sixfold higher risk of developing cervical cancer than those uninfected with HIV,^3^ which has been defined as acquired immune deficiency syndrome (AIDS)‐defining disease.^4^ WHO has prioritized evidence‐based interventions for WLHIV including integrated vaccination, screening, and treatment services, with the call for action toward cervical cancer elimination in 2018.^5^

Currently, screening is still the main method for cervical cancer prevention since the unavailability of HPV vaccine for many women, especially those living in low‐resource settings. Cytology, HPV test, and visual inspections with acetic acid (VIA) have been recommended for cervical cancer primary screening among general women.^6^, ^7^ With the high sensitivity,^8^ good reproducibility, and long‐term reassurance after a negative test result^9^ for HPV test, more and more countries are switching from cytology‐based to HPV‐based screening. However, managing HPV‐positive women to improve the relatively lower specificity is vital for maximizing the screening benefits while reducing unnecessary colposcopy and overtreatment. In published global guidelines and Chinese guideline, VIA, cytology, as well as HPV‐16/18 with reflex cytology have been recommended to triage HPV‐positive individuals in general women.^6^, ^7^, ^10^ Triage with HPV‐16/18 alone,^11^ or HPV‐16/18/31/33/45 genotyping^12^ have also been evaluated among Chinese general women with optimal performance in previous studies. These primary and triage methods are also recommended among WLHIV with shorter screening interval.^6^, ^7^, ^13^ However, the screening evidence for WLHIV is insufficient globally and these strategies have not been validated among Chinese WLHIV. Besides the condition of very limited screening guidelines for WLHIV, regarding to the changing demographic characteristics of WLHIV in cART era, these guidelines also need further updates. Clinical performance influenced by cART duration, CD4 count, and age also needs to be explored to provide specific screening guidance for WLHIV.

Thus, we conducted a study to evaluate the clinical performance of primary screening with HPV tests, cytology, and VIA or visual inspections with Lugol's iodine (VIA/VILI) and several triage strategies for HPV‐positive individuals among WLHIV in a high HIV epidemic and low‐resource area in China. Clinical performance of primary screening was further evaluated by the stratification on CD4 count, cART duration, and age.

## 2 METHODS

### 2.1 Study population

**FIGURE 1:**
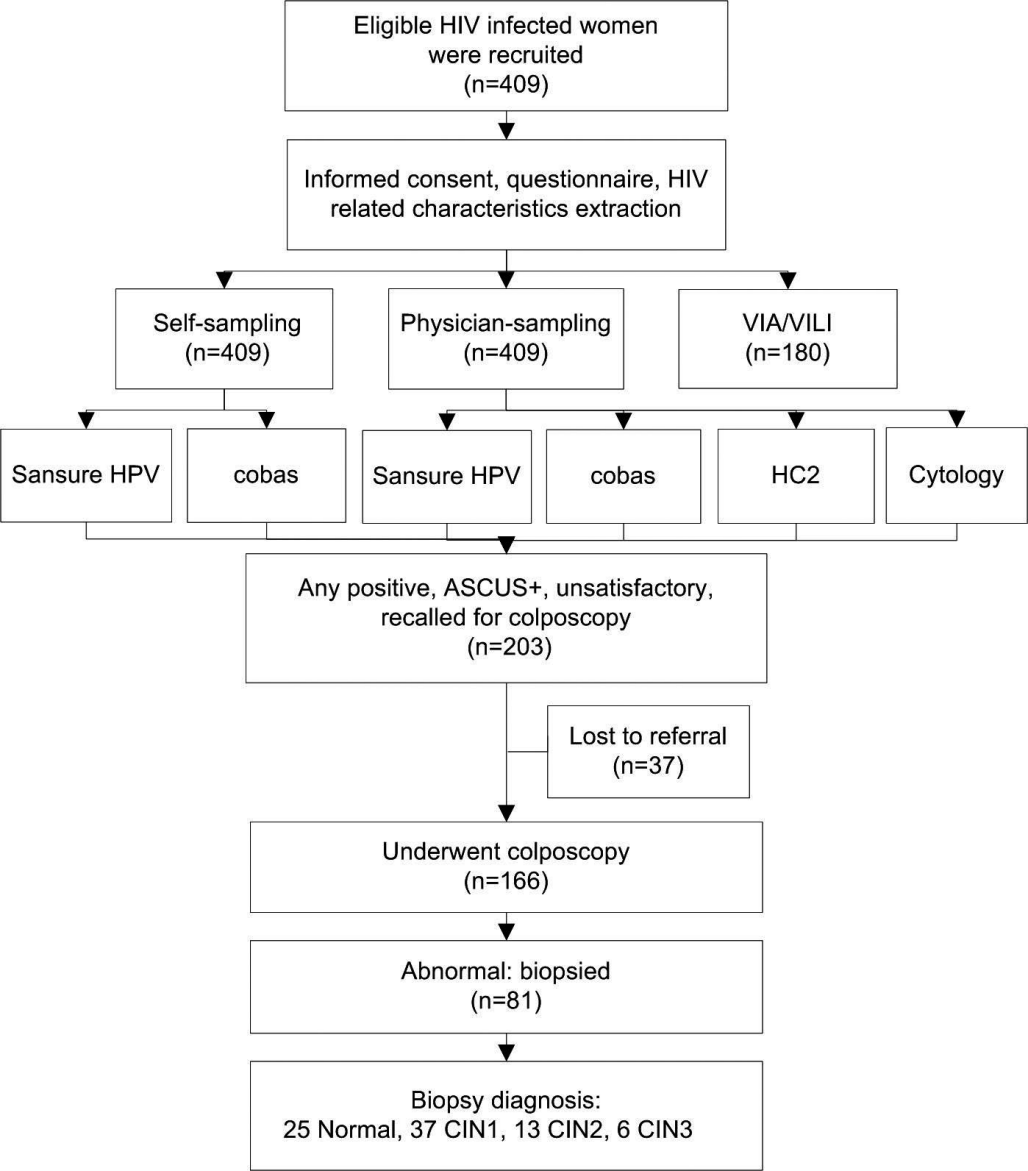
Flowchart of study procedure. ASCUS+, atypical squamous cells of undetermined significance or worse; CIN, cervical intraepithelial neoplasia, grade 1 (CIN1), grade 2 (CIN2), grade 3 (CIN3); VIA/VILI, visual inspection with acetic acid (VIA) or Lugol's iodine (VILI)

Participants were recruited from the Department of AIDS antiretroviral therapy at the Third People's Hospital of Kunming, Yunnan province of China form January 2019 to August 2020. Inclusion criteria were: (1) HIV seropositive and with ongoing cART, (2) aged 18 years and older, (3) had initiated sexual activity, (4) able to both physically and mentally undergo cervical sampling, and (5) had an intact cervix and were no currently pregnant. After informed consent, participants were interviewed individually by a trained health worker for demographic characteristics. HIV‐related characteristics were extracted from medical records. The study design is illustrated in Figure 1.

### 2.2 Specimen collection and VIA/VILI

Women collected one vaginal self‐sample with vaginal brush (Qiagen) under the instruction of healthcare providers, and placed at PreservCyt solution (ThinPrep, Hologic). Two exfoliated cervical cell samples were then collected by a trained gynecologist and placed at PreservCyt solution and hybrid capture 2 (HC2) liquid medium (Qiagen), respectively. VIA/VILI was performed on 180 women after sample collection by the trained gynecologist following the International Agency for Research on Cancer (IARC) guidelines.^14^

### 2.3 Laboratory testing

All collected samples were placed at 4° refrigerator and transferred to KingMed diagnostic laboratory. Self‐collected and the first physician‐collected samples were prepared for Sansure HPV testing^®^ (Sansure Biotech) and cobas 4800 HPV testing^®^ (Roche Diagnostics). The second physician‐collected samples were prepared for Digene HC2 HPV testing^®^ (Qiagen). Residuals of the first physician‐collected samples were processed for cytology slides and interpreted by experienced cytologists.

Sansure HPV test^®^ is PCR‐based pioneered One‐step Fast Release technology, using real‐time fluorescent quantitative PCR to target 15 HPV types, including 13 high‐risk types: HPV‐16/18/31/33/35/39/45/51/52/56/58/59/68, and 2 possibly high‐risk types: HPV‐53 and 66, and has been approved by the European Union Certificate. HPV status is measured by the cycle numbers observed (Ct) when the fluorescent signal reaches the set type‐specific threshold. A Ct ≤39 is considered HPV‐positive and a Ct >39 is considered negative.^11^ The Roche cobas 4800 HPV test detects 14 HPV types (the 13 high‐risk types and HPV‐53) with specific genotyping for HPV‐16 and HPV‐18. The cobas 4800 system fully automates the sample preparation with real‐time PCR technology for amplification and detection, with β‐globulin gene working as the internal control. Digene HC2 HPV testing is based on hybridization of HPV DNA with a high‐risk RNA probe cocktail that collectively targets 13 high‐risk HPV types but does not discriminate individual genotypes.

Cytology results were reported according to the Bethesda 2014 classification system: negative for intraepithelial lesion or malignancy (NILM), atypical squamous cells of undetermined significance (ASC‐US), atypical squamous cells cannot exclude HSIL (ASC‐H), atypical glandular cells, low‐grade squamous intraepithelial lesion (LSIL), high‐grade squamous intraepithelial lesion (HSIL), squamous cell carcinoma (SCC), adenocarcinoma in situ (AIS), and adenocarcinoma (ADC).

Women positive for any HPV or with cytological atypical squamous cells of undetermined significance or worse (ASCUS+) or unsatisfactory were recalled for colposcopy examination, and biopsied if any abnormality was identified. Endocervical curettage was performed if the squamocolumnar junction was invisible. Biopsy tissues were immediately immersed in 10% buffered formalin and transported to KingMed diagnostics for processing and diagnosis by experienced pathologists who were blinded to other screening results. Pathology results were reported as normal, cervical intraepithelial neoplasia grade 1 (CIN1), grade 2 (CIN2), grade 3 (CIN3), microinvasive carcinoma (MIC), SCC, AIS, and ADC. Women with CIN2 or worse (CIN2+) were recommended for treatment according to clinical guidelines. Women who were negative for all tests (cytology < ASCUS and no HPV detected) were considered to be negative for the outcome of CIN2+.

### 2.4 Statistical analysis

Clinical performance of primary screening and triage strategies was evaluated with sensitivity, specificity, positive predictive value (PPV), negative predictive value, area under the curve (AUC), detection rate, and colposcopy referral. Relative sensitivity, specificity, detection rate, and colposcopy referral (reference for primary screening: liquid‐based cytology [LBC] [ASCUS+], reference for triage strategies: HPV test without triage) were also calculated with 95% confidence interval (95% CI). Significant difference was considered if the 95% CI of the relative values was entirely above or below one. Sensitivity and specificity were further stratified by duration of cART, CD4 count, and age and were compared using Chi‐square tests and logistic regression. Sensitivity or specificity found to be statistically different for varying strata in univariate analysis was included into multivariate analysis. Data were analyzed on SPSS 20.0 and R software 3.6.2.

We evaluated the following primary screening and triage strategies:

1. LBC
  1.1 LBC (ASCUS+)
  1.1 LBC (LSIL+)
2. HPV tests
  2.1 HC2
  2.2 Triage of HC2‐positive women
    2.2.1 LBC (ASCUS+)
    2.2.2 LBC (LSIL+)
    2.2.3 VIA/VILI
  2.3 Physician‐cobas HPV
  2.4 Triage of physician‐cobas HPV‐positive women
    2.4.1 LBC (ASCUS+)
    2.4.2 LBC (LSIL+)
    2.4.3 HPV‐16/18 genotyping
    2.4.4 HPV‐16/18 genotyping with reflex LBC (ASCUS+) for those without HPV‐16/18
    2.4.5 VIA/VILI
  2.5 Self‐cobas HPV
  2.6 Triage of self‐cobas HPV‐positive women
    2.6.1 HPV‐16/18 genotyping
  2.7 Physician‐Sansure HPV
  2.8 Triage of physician‐Sansure HPV‐positive women
    2.8.1 LBC (ASCUS+)
    2.8.2 LBC (LSIL+)
    2.8.3 HPV‐16/18 genotyping
    2.8.4 HPV‐16/18/31/33/45/52/58 genotyping
    2.8.5 HPV‐16/18 genotyping with reflex LBC (ASCUS+) for those without HPV‐16/18
    2.8.6 VIA/VILI
  2.9 Self‐Sansure HPV
  2.10 Triage of self‐Sansure HPV‐positive women
    2.10.1 HPV‐16/18 genotyping
    2.10.2 HPV‐16/18/31/33/45/52/58 genotyping
3. VIA/VILI

## 3 RESULTS

### 3.1 Study population

Of 409 recruited women, 37 women lost to follow‐ups for colposcopy. Three hundred and seventy‐two women with adequate outcomes were included into the final analysis. The median age was 40 (35–47) years, 60.2% have been married, 71.2% had education at junior high school or below, 48.1% were unemployed, and 42.7% were farmer or rural migrant worker. Median age at sexual debut and first delivery was 20 (19–22) and 24 (22–27) years, respectively. The median years living with HIV and duration on cART was 5 (2–9) and 4 (2–8) years. The median values of nadir (at cART initiation) and current CD4 count were 237 (IQR 137–326) and 550 (IQR 401–704) cells/µl, the initial (at cART initiation) and current HIV viral loads were 50 (IQR <50–14,012) and <50 (IQR <50 to <50) copies/ml, respectively. Data are shown in Table 1.

**TABLE 1:** Demographical and HIV‐related characteristic of WLHIV

Characteristics	*N* or median^a^	% or IQR^a^	Characteristics	*N* or median^a^	% or IQR^a^
Age, years old	40^a^	35–47^a^	Years living with HIV	5^a^	2–9^a^
≥40	204	54.8	≤2	96	25.8
<40	168	45.2	3–9	171	46
Ethnicity			≥10	48	12.9
Han	312	83.9	Unknown	57	15.3
Minority	60	16.1	cART duration time, years	4^a^	2–8^a^
Marriage status			>2	206	55.4
Unmarried	14	3.8	≤2	107	28.8
Married	224	60.2	Unknown	59	15.9
Divorced/widowed	134	36.0	Nadir CD4 count, cells/µl	237^a^	137–326^a^
Education			≥200	208	55.9
Senior high school or above	107	28.8	<200	125	33.6
Junior high school	143	38.4	Unknown	39	10.5
Primary school or below	122	32.8	Current CD4 count, cells/µl	550^a^	401–704^a^
Occupation			≥350	274	81.3
Public institution/enterprises	34	9.1	<350	63	18.7
Farmer/rural migrant worker	159	42.7	Unknown	35	9.4
Unemployed	179	48.1	Initial HIV VL, copies/ml	50^a^	<50–14,012^a^
Age of sexual debut	20^a^	19–22^a^	<1000	182	48.9
Age at first delivery	24^a^	22–27^a^	≥1000	95	25.5
Currently contraception			Unknown	95	25.5
Condom	171	46.0	Current HIV VL, copies/ml	<50^a^	<50–<50^a^
Oral contraceptive	16	4.3	<50	320	86.0
Intrauterine device	83	22.3	≥50	12	3.2
Tubal ligation	24	6.5	Unknown	40	10.8
No sexual behavior	78	21.0			

Abbreviations: cART, combination antiretroviral therapy; IQR, inter‐quartile range; VL, viral load; WLHIV, women living with HIV.

^a^ Median and IQR.

### 3.2 Clinical performance of the primary screening approaches

#### 3.2.1 Performance of LBC, HPV tests, and VIA/VILI

Positivity of LBC (ASCUS+), LBC (LSIL+), HC2, cobas, Sansure HPV, and VIA/VILI was 6.5%, 4.1%, 23.7%, 18.7%, 31.7%, and 23.2%, respectively (Table S1). In comparison to LBC (ASCUS+) (sensitivity: 61.1%, specificity: 96.3%), HPV tests demonstrated higher sensitivity (HC2: 100%, ratio: 1.64, 95% CI: 1.21–2.80; cobas: 89.5%, 1.45, 1.12–2.42; Sansure HPV: 100%, 1.64, 1.21–2.80) but lower specificity (HC2: 80.4%, ratio: 0.83, 95% CI: 0.79–0.87; cobas: 85.1%, 0.88, 0.84–0.92; Sansure HPV: 72.0%, 0.75, 0.70–0.79), while VIA/VILI showed lower but non‐significant sensitivity (40.0%, ratio: 1.00, 95% CI:1.00–6.32), and inferior specificity (77.3%, 0.80, 0.73–0.86) for detecting CIN2+ (Table 2, Table S2). AUC was 0.79, 0.90, 0.87, 0.86, and 0.59 for LBC, HC2, cobas, Sansure HPV, and VIA/VILI, respectively, at CIN2 threshold (Table 2). HPV test and VIA/VILI need more colposcopy referral compared to LBC (ASCUS+) (ratio: 3.54, 2.83, 4.83, and 4.88 for HC2, cobas, Sansure HPV, and VIA/VILI, respectively) (Table 2, Table S2). The accuracy for detecting CIN3+ showed similar trend comparing HPV with LBC (Tables S3 and S4).

**TABLE 2:** Clinical performance of primary screening methods in WLHIV for the detection of CIN2+

Methods	Sensitivity% (95% CI)	Specificity% (95% CI)	PPV% (95% CI)	NPV% (95% CI)	AUC (95% CI)	Referral rate% (95% CI)
Physician‐sampling						
LBC (ASCUS+)	61.1 (35.7–82.7)	96.3 (93.8–98.0)	45.8 (25.6–67.2)	98.0 (95.9–99.2)	0.79 (0.74–0.83)	6.5 (4.4–9.5)
LBC (LSIL+)	38.9 (17.3–64.3)	97.7 (95.6–99.0)	46.7 (21.3–73.4)	96.9 (94.5–98.4)	0.68 (0.63–0.73)	4.1 (2.5–6.6)
HC2	100 (81.5–100)	80.4 (75.7–84.4)	21.2 (13.1–31.4)	100 (98.7–100)	0.90 (0.87–0.93)	23.7 (19.6–28.3)
Cobas	89.5 (66.9–98.7)	85.1 (81.0–88.7)	24.6 (15.1–36.5)	99.3 (97.6–99.9)	0.87 (0.84–0.91)	18.7 (15.1–23.0)
Sansure HPV	100 (82.4–100)	72.0 (67.0–76.6)	16.1 (10.0–24.0)	100 (98.6–100)	0.86 (0.82–0.89)	31.7 (27.2–36.6)
VIA/VILI	40.0 (5.3–85.3)	77.3 (70.1–83.5)	5.1 (0.6–17.3)	97.7 (93.4–99.5)	0.59 (0.51–0.66)	12.5 (8.3–18.4)
Self‐sampling						
Cobas	100 (81.5–100)	71.9 (66.8–76.6)	15.8 (9.6–23.8)	100 (98.5–100)	0.86 (0.82–0.89)	31.7 (27.1–36.6)
Sansure HPV	100 (82.4–100)	66.9 (61.7–71.7)	14.0 (8.6–21.0)	100 (98.4–100)	0.83 (0.79–0.87)	36.6 (31.8–41.6)

Abbreviations: ASCUS+, atypical squamous cells of undetermined significance or worse; AUC, area under the curve; CI, confidence interval; CIN2+, cervical intraepithelial neoplasia grade 2 or worse; HC2, hybrid capture 2; LBC, liquid‐based cytology; LSIL, low‐grade squamous intraepithelial lesions or worse; NPV, negative predictive value; PPV, positive predictive value; VIA/VILI, visual inspection with acetic acid (VIA) or Lugol's iodine (VILI); WLHIV, women living with HIV.

#### 3.2.2 Performance of self‐ and physician‐HPV tests

Positivity of self‐HPV was 31.7% for cobas and 36.6% for Sansure HPV. For CIN2+ detection, cobas self‐HPV demonstrated a slightly higher sensitivity (ratio: 1.12, 95% CI: 1.01–1.53) but lower specificity (0.85, 0.80–0.89) with similar AUC but higher colposcopy referral rate (1.69, 1.46–2.01) compared to physician‐HPV (Table 2, Table S2). For Sansure HPV test, comparable sensitivity, specificity, AUC, and colposcopy referral were identified between self‐ and physician‐sampling (Table 2, Table S2). Similar performance was identified for CIN3+ detection (Tables S3 and S4).

#### 3.2.3 Performance of Sansure HPV compared with cobas and HC2

Compared with cobas, Sansure HPV had a marginally increased sensitivity (ratio: 1.12, 95% CI: 1.01–1.50), lower specificity (0.84, 0.80–0.88), and similar AUC, with higher colposcopy referral (1.71, 1.48–2.04) in detecting CIN2+ (Table 2, Table S2). While Sansure HPV showed comparable clinical performance with HC2 (ratio: 1.00, 95% CI: 1.00–1.23 for sensitivity; 0.90, 0.86–0.93 for specificity; 1.33, 1.21–1.51 for referral rate). The results for detecting CIN3+ were similar to that of CIN2+ (Tables S3 and S4).

### 3.3 Clinical performance of triage strategies for HPV‐positive women

#### 3.3.1 HPV tests with triage of LBC

Among HPV‐positive women, the test positivity of triage strategies is shown in Table S5. Compared with HPV test alone, LBC (ASCUS+) triage achieved 15%–35% elevated specificity (ratio: 1.21, 95% CI: 1.16–1.28 for HC2; 1.15, 1.11–1.20 for cobas; 1.35, 1.28–1.45 for Sansure HPV) and increased PPV (ratio: 2.60, 95% CI: 1.80–3.74 for HC2; 2.35, 1.65–3.36 for cobas; 3.42, 2.33–5.00 for Sansure HPV), at a cost of approximately 40% declined sensitivity and slight reduction of AUC for detecting CIN2+, with decreased colposcopy referral (Table 3, Table S6). Similar clinical performance was identified for detecting CIN3+ (Tables S7 and S8). With LBC (LSIL+) triage, specificity and PPV increased and referral rate declined while large reduction on the sensitivity and AUC at CIN 2 and CIN3+ threshold (Table 3, Tables S6–S8).

**TABLE 3:** Clinical performance of triage strategies in HPV‐positive WLHIV for the detection of CIN2+

Strategies	Sensitivity% (95% CI)	Specificity% (95% CI)	PPV% (95% CI)	NPV% (95% CI)	AUC% (95% CI)	Referral rate% (95% CI)
Physician‐sampling						
For HC2‐positive						
LBC (ASCUS+)	61.1 (35.7–82.7)	97.4 (95.0–98.8)	55.0 (31.5–76.9)	97.9 (95.8–99.2)	0.79 (0.75–0.83)	5.6 (3.6–8.5)
LBC (LSIL+)	38.9 (17.3–64.3)	98.2 (96.2–99.4)	53.8 (25.1–80.8)	96.8 (94.4–98.4)	0.69 (0.64–0.73)	3.6 (2.1–6.1)
VIA/VILI	40.0 (5.3–85.3)	94.7 (89.8–97.7)	20.0 (2.5–55.6)	97.9 (94.1–99.6)	0.67 (0.59–0.75)	6.0 (3.3–10.6)
For cobas‐positive						
HPV‐16/18	68.4 (43.4–87.4)	96.3 (93.7–98.0)	50.0 (29.9–70.1)	98.3 (96.2–99.4)	0.82 (0.78–0.86)	7.0 (4.9–10.1)
LBC (ASCUS+)	57.9 (33.5–79.7)	97.7 (95.5–99.0)	57.9 (33.5–79.7)	97.7 (95.5–99.0)	0.78 (0.73–0.82)	5.1 (3.3–7.9)
LBC (LSIL+)	36.8 (16.3–61.6)	98.6 (96.7–99.5)	58.3 (27.7–84.8)	96.6 (94.2–98.3)	0.68 (0.63–0.73)	3.3 (1.9–5.6)
HPV‐16/18|LBC (ASCUS+)	78.9 (54.4–93.9)	94.6 (91.7–96.7)	44.1 (27.2–62.1)	98.8 (97.0–99.7)	0.87 (0.83–0.90)	9.2 (6.7–12.6)
VIA/VILI	40.0 (5.3–85.3)	96.3 (92.1–98.6)	25.0 (3.2–65.1)	98.1 (94.6–99.6)	0.68 (0.61–0.75)	4.8 (2.4–9.1)
For Sansure HPV‐positive						
HPV‐16/18	68.4 (43.4–87.4)	94.9 (92.1–97.0)	41.9 (24.5–60.9)	98.2 (96.2–99.4)	0.82 (0.77–0.86)	8.3 (5.9–11.6)
HPV‐16/18/31/33/45/52/58	89.5 (66.9–98.7)	81.9 (77.4–85.7)	21.0 (12.7–31.5)	99.3 (97.5–99.9)	0.86 (0.82–0.89)	21.8 (17.9–26.2)
LBC (ASCUS+)	57.9 (33.5–79.7)	97.5 (95.2–98.8)	55.0 (31.5–76.9)	97.7 (95.6–99.0)	0.78 (0.73–0.82)	5.4 (3.5–8.2)
LBC (LSIL+)	36.8 (16.3–61.6)	98.3 (96.3–99.4)	53.8 (25.1–80.8)	96.7 (94.2–98.3)	0.68 (0.63–0.72)	3.5 (2.1–5.9)
HPV‐16/18|LBC (ASCUS+)	78.9 (54.4–93.9)	92.9 (89.7–95.4)	37.5 (22.7–54.2)	98.8 (96.9–99.7)	0.86 (0.82–0.89)	10.8 (8.0–14.3)
VIA/VILI	40.0 (5.3–85.3)	94.5 (89.8–97.4)	18.2 (2.3–51.8)	98.1 (94.5–99.6)	0.67 (0.60–0.74)	6.5 (3.7–11.4)
Self‐sampling						
For cobas‐positive						
HPV‐16/18	72.2 (46.5–90.3)	92.4 (89.1–95.0)	33.3 (19.1–50.2)	98.4 (96.4–99.5)	0.82 (0.78–0.86)	10.8 (8.0–14.5)
For Sansure HPV‐positive						
HPV‐16/18	73.7 (48.8–90.9)	94.3 (91.4–96.5)	41.2 (24.6–59.3)	98.5 (96.6–99.5)	0.84 (0.80–0.88)	9.1 (6.6–12.5)
HPV‐16/18/31/33/45/52/58	94.7 (74.0–99.9)	77.3 (72.6–81.6)	18.4 (11.3–27.5)	99.6 (98.0–100)	0.86 (0.82–0.89)	26.3 (22.1–31.0)

Abbreviations: ASCUS+, atypical squamous cells of undetermined significance or worse; AUC, area under the curve; CI, confidence interval; CIN2+, cervical intraepithelial neoplasia grade 2 or worse; HC2, hybrid capture 2; LBC, liquid‐based cytology; LSIL+, low‐grade squamous intraepithelial lesions or worse; NPV, negative predictive value; PPV, positive predictive value; VIA/VILI, visual inspection with acetic acid (VIA) or Lugol's iodine (VILI); WLHIV, women living with HIV.

#### 3.3.2 HPV tests with triage of VIA/VILI

Compared to HPV test without triage, 8%–32% specificity was elevated (ratio: 1.17, 95% CI: 1.10–1.27 for HC2, 1.08, 1.04–1.14 for cobas, 1.32, 1.21–1.46 for Sansure HPV) with insignificant change on PPV and AUC by VIA/VILI triage, while the sensitivity declined more than 50% (ratio: 0.40–0.50), with largely decreased colposcopy referral (ratio: 0.22–0.38) (Table 3, Table S6).

#### 3.3.3 HPV genotyping triage

Compared with HPV primary screening, triage with HPV‐16/18 genotyping demonstrated 13%–32% increased specificity (ratio: 1.13, 95% CI: 1.09–1.18 for cobas; 1.32, 1.25–1.41 for Sansure HPV) and elevated PPV (2.03, 1.50–2.75 for cobas; 2.60, 1.88–3.61 for Sansure HPV), with approximately 30% reduced sensitivity and a mild decrease on AUC at CIN2+ threshold (Table 3, Table S6). HPV‐16/18/31/33/45/52/58 genotyping triage increased 14% specificity (ratio: 1.14, 95% CI: 1.09–1.20) and improved PPV (1.30, 1.10–1.54), aligning with 11% decreased sensitivity and equivalent AUC for detecting CIN2+ (Table 3, Table S6). HPV‐16/18 genotyping with reflex LBC (ASCUS+) for those without HPV‐16/18 triage elevated 11%–29% of specificity (ratio: 1.11, 95% CI: 1.07–1.16 for cobas; 1.29, 1.22–1.38 for Sansure HPV) and increased PPV (1.79, 1.40–2.29 for cobas; 2.33, 1.76–3.07 for Sansure HPV), with 12%–21% of sensitivity reduction and equivalent AUC for detecting CIN2+ (Table 3, Table S6). The genotyping triage and HPV‐16/18 with reflex LBC largely reduced the colposcopy referral at CIN2 threshold (Table 3, Table S6). The triaging approaches achieved comparable performance for CIN3+ detection (Tables S7 and S8). Self‐HPV test with HPV‐16/18 or HPV‐16/18/31/33/45/52/58 genotyping triage showed similar trend with physician‐HPV test (Table 3, Tables S6–S8).

### 3.4 Clinical performance of primary screening approaches stratified by CD4 count, cART duration, and age

For CIN2+ detection, specificity of LBC (ASCUS+) (91.2% vs. 97.3%, *p* < 0.05) and HC2 (69.1% vs. 85.5%, *p* < 0.05) was decreased among women with lower current CD4 count (<350 cells/µl) (Table 4). Among those who underwent short duration of cART (≤2 years), declined specificity of HC2 (69.4% vs. 87.5%, *p* < 0.001), cobas (77.8% vs. 90.3%, *p* < 0.05), and Sansure HPV (56.0% vs. 80.1%, *p* < 0.001) was observed (Table 4). Improved specificity of Sansure HPV (78.0% vs. 67.0%, *p* < 0.05) was observed among women aged <40 years (Table 4). No significant difference in sensitivities was identified with the stratification of CD4 count, cART duration, and the age (Table 4). CD4 count, cART duration, and age were included into univariate and multivariate logistic regression analyses, and only cART duration <2 years was associated with reduced specificity of HC2 (aOR: 1.87, 95% CI: 1.22–3.91) and Sansure HPV (2.48, 1.43–4.29) significantly (Table S9).

**TABLE 4:** Clinical performance of screening methods stratified by CD4 count, cART duration, and age in WLHIV for the detection of CIN2+

Strata	*N*	CIN2+	LBC (ASCUS+)		HC2		Cobas		Sansure HPV		*N*	CIN2+	VIA/VILI	
			SE	SP	SE	SP	SE	SP	SE	SP			SE	SP
CD4 count														
≥350 cell/µl	274	10	80.0	97.3	100	85.5	90.0	87.4	100	75.8	131	3	33.3	78.7
<350 cell/µl	63	6	50.0	91.2	100	69.1	100	82.5	100	63.2	30	1	0	64.3
*p*			0.30	**0.04**	—	**0.01**	1.00	0.39	—	0.07			1.00	0.14
cART duration														
>2 years	206	10	80.0	97.4	100	87.5	100	90.3	100	80.1	93	3	66.7	76.7
≤2 years	107	7	42.9	93.9	100	69.4	85.7	77.8	100	56.0	42	1	0	75.6
*p*			0.16	0.19	—	**0.0001**	0.41	**0.01**	—	**0.0001**			1.00	1.00
Age														
≥40 years	204	10	55.6	97.9	100	76.7	90.0	82.9	100	67.0	89	2	50.0	81.4
<40 years	168	9	66.7	94.3	100	84.9	88.9	87.9	100	78.0	80	3	33.3	72.7
*p*			0.65	0.09	—	0.07	1.00	0.23	—	**0.02**			1.00	0.20

Bold text highlights statistical significance.

—: *p* was not calculated due to similar compared variables.

Abbreviations: ASCUS+, atypical squamous cells of undetermined significance or worse; cART, combination antiretroviral therapy; CIN2+, cervical intraepithelial neoplasia grade 2 or worse; HC2, hybrid capture 2; LBC, liquid‐based cytology; SE, sensitivity; SP, specificity; VIA/VILI, visual inspection with acetic acid (VIA) or Lugol's iodine (VILI); WLHIV, women living with HIV.

## 4 DISCUSSION

For primary screening approaches, HPV test was highly sensitive and moderately specific. LBC (ASCUS+) demonstrated inferior sensitivity while highest specificity, VIA/VILI showed the lowest sensitivity and specificity. For triage strategies for HPV‐positive women, VIA/VILI triage showed unsatisfactory performance, HPV‐16/18 genotyping demonstrated comparable performance to LBC (ASCUS+) triage, while HPV‐16/18/31/33/45/52/58 and HPV‐16/18 with reflex LBC achieved optimal clinical accuracy. Specificity of HPV tests was declined among women with short duration of cART.

Successful cervical cancer control has been achieved by organized cytology screening in high‐income countries. In China, cytology is still the main primary screening method for national cervical cancer screening since 2009 for general women. LBC (ASCUS+) in this study showed inferior sensitivity and similar specificity compared to Chinese general women (80.7% and 94.0%),^15^ lower sensitivity but higher specificity to WLHIV in Africa (76.7% and 77.3%),^16^ while comparable accuracy with WLHIV in India (63.3% and 94.5%).^17^ Due to the low sensitivity and the variable diagnostic accuracy, LBC would not be highly recommended for primary screening among Chinese WLHIV except the high‐quality LBC is available. Visual inspection is simple and affordable for low‐ and middle‐income countries (LMICs), whereas suboptimal performance was identified in our study. VIA/VILI showed lower sensitivity and specificity compared to Chinese general women (50.3% and 87.4%)^15^ and WLHIV in India (90.9% and 84.9%),^17^ while lower sensitivity and a slightly higher specificity for WLHIV in Africa (61.5% and 71.9%).^16^ VIA/VILI, therefore, was not recommended for WLHIV in China except other algorithms is limited.

HPV test in this study showed similar sensitivity but lower specificity compared to that of in Chinese general women (97.0% and 82.7%, HC2^15^; 96.7% and 82.1%, Sansure HPV^11^), while both sensitivity and specificity were higher than that of among WLHIV in Africa (88.8% and 55.4%, HC2)^18^ and India (94.6% and 77.4%, HC2).^17^ Of note, PCR‐based self‐HPV showed comparable screening performance with physician‐HPV in this study. Self‐HPV test revealed the prospect to improve screening coverage, reduce health disparity to benefit women with limited access to healthcare services,^19^ decrease stigma among WLHIV, and the risk for HIV infection of health workers and thus would more applicable for this population. A large‐scale randomized clinical trial in China has demonstrated the effectiveness of high‐risk HPV test as primary screening method in primary healthcare centers.^20^ In China, HPV assay is running on the fast lane of development, as of 2020, hundreds of HPV detection products have been approved by the National Medical Products Administration. Affordable, sensitive, user‐friendly, rapid, and robust “point‐of‐care” HPV technology is now under validation. Notably, domestic PCR‐HPV in our study showed similar clinical performance to the FDA‐approved cobas and HC2 at lower cost and shorter detection time. HPV test is, therefore, expected to be the option for WLHIV in the future in China even other LMICs.

However, the specificity of HPV test needs to be improved for effective cervical cancer screening. In our study, VIA/VILI triage for HPV‐positive individuals showed lower sensitivity but higher specificity for WLHIV in Africa (62.9% and 65.7%),^16^ it was not recommended due to large reduction in sensitivity. LBC (ASCUS+) triage showed lower sensitivity and higher specificity for Chinese general women (83.7% and 65.8%)^15^ and WLHIV in Africa (75.9% and 63.8%).^16^ HPV‐16/18 genotyping triage is inferior for Chinese general women (sensitivity: 73.6%, specificity: 97.0%, Sansure HPV),^11^ and it demonstrated comparable efficacy to LBC triage. While triage on restricted specific popular HPV genotypes in our study (HPV‐16/18/31/33/45/52/58) demonstrated the optimal clinical performance, with similar sensitivity but lower specificity for Chinese general women (90.1% and 89.5%, Sansure HPV).^11^ The two‐tests triage (HPV‐16/18 with reflex LBC) showed lower sensitivity but higher specificity compared to Chinese general women (98.0%, 57.0%, PCR‐HPV)^12^ and WLHIV in the United States (84.0% and 78.0%),^21^ and the clinical accuracy was similar to the restricted HPV genotyping triage in this study. Considering primary HPV test with restricted genotyping triage could be achieved within one test, it is highly recommended for WLHIV in China compared to the two‐tests triage strategy. While HPV‐16/18 genotyping or high‐quality LBC triage is recommended if there is limited accessibility to the restricted genotyping.

Immunosuppression may affect the screening accuracy among WLHIV. Similar with African WLHIV,^22^ short duration on cART was significantly associated with decreased HPV specificity in our study. Consecutive and longer cART may reverse the immunosuppression, while short duration of cART users may represent worse immunosuppression. Thus, the specificity of HPV test maybe lower in these women due to the higher prevalence of transient or non‐clinically relevant HPV infections. CD4 cell count of 350 cells/ml or less was associated with reduced specificity on cytology^23^ and HPV.^22^, ^24^ However, CD4 count was observed affecting none of the test in this study maybe due to limited sample size. Further evaluation is suggested to evaluate the screening performance among women with different CD4 counts. When HPV test was applied among individuals with short cART duration and severe immunodeficiency, the optimal triage algorithm is recommended to elevate the screening specificity.

To the best of our knowledge, this study is first of its kind evaluating the clinical performance of multiple cervical cancer screening strategies concurrently among Chinese WLHIV, including LBC, hybrid capture‐ and PCR‐based HPV tests, and visual inspections, as single or consecutive triage algorithms, by physician‐ and self‐sampling. In addition, stratification analyses of clinical accuracy on CD4 count, cART duration, and age were conducted to provide the evidence for specific screening guidance. Furthermore, our study has demonstrated the potentiality of novel self‐Sansure PCR HPV test serving as a low‐cost alternative for future “point‐of‐care” algorithm among WLHIV. The major limitation of the study is the limited sample size, and not all participants were provided with VIA/VILI inspections.

In conclusion, HPV test is highly recommended for primary cervical cancer screening among WLHIV in China, while high‐quality cytology is recommended if HPV test is not available. For HPV‐positive triage, HPV‐16/18/31/33/45/52/58 was highly recommended, and HPV‐16/18 with reflex LBC cytology triage is recommended if HPV genotyping is not feasible. The affordable one‐time self‐HPV test with the restricted genotyping triage would be the most feasible and optimal cervical cancer screening strategy for WLHIV in China, especially in high HIV epidemic settings. Large‐scale prospective study is suggested to supplement evidence on clinical performance of screening strategies, and address the initiated age of screening and screening intervals for WLHIV.

## CONFLICT OF INTEREST

The authors have no conflicts of interest to declare.

## ETHICAL APPROVAL STATEMENT

The study protocol was approved by the Ethical Review Committees of National Cancer Center/Cancer Hospital, Chinese Academy of Medical Sciences and Peking Union Medical College, Beijing, China.

Data availability statement The data that support the findings of this study are available from the corresponding author upon reasonable request.

## Supporting information

Tables S1–S9

## Data Availability

Retrieved from the SEER* STAT database according to the SEER research data usage protocol.
