# Supplementary material for: Performance of cervical cancer screening and triage strategies among women living with HIV in China
Source: Cancer Med. 2021 Aug 2;10(17):6078–88. doi: 10.1002/cam4.4152 (PMC8419757; doi:10.1002/cam4.4152)
Supplement: Supplementary file 1 — Tables S1–S9 [file CAM4-10-6078-s001.docx]

| **Supplementary 1 Basic information for primary screening methods in WLHIV** | | | | | | | | | | | | | |
| --- | --- | --- | --- | --- | --- | --- | --- | --- | --- | --- | --- | --- | --- |
| **Programs** | **No. screened** | **No. screen positive** | **%** | **No. with colposcopy** | **No. with abnormal colposcopy** | **%** | **No. CIN1** | **%** | **No. CIN2+** | **%** | **No. CIN3+** | **%** |  |
| **Total** | 372 | 170 | 45.7 | 170 | 81 | 47.6 | 37 | 9.9 | 19 | 5.1 | 6 | 1.6 |  |
| **Physician-sampling** |  |  |  |  |  |  |  |  |  |  |  |  |  |
| LBC (ASCUS+) | 370 | 24 | 6.5 | 24 | 16 | 66.7 | 6 | 1.6 | 12 | 3.2 | 6 | 1.6 |  |
| LBC (LSIL+) | 370 | 15 | 4.1 | 15 | 9 | 60.0 | 4 | 1.1 | 7 | 1.9 | 4 | 1.1 |  |
| HC2 | 359 | 85 | 23.7 | 85 | 54 | 63.5 | 21 | 5.8 | 18 | 5.0 | 6 | 1.7 |  |
| cobas | 369 | 69 | 18.7 | 69 | 49 | 71.0 | 20 | 5.4 | 17 | 4.6 | 6 | 1.6 |  |
| Sansure HPV | 372 | 118 | 31.7 | 118 | 65 | 55.1 | 24 | 6.5 | 19 | 5.1 | 6 | 1.6 |  |
| VIA/VILI | 168 | 39 | 23.2 | 21 | 10 | 47.6 | 6 | 3.6 | 2 | 1.2 | 0 | 0 |  |
| **Self-sampling** |  |  |  |  |  |  |  |  |  |  |  |  |  |
| cobas | 360 | 114 | 31.7 | 114 | 63 | 55.3 | 27 | 7.5 | 18 | 5.0 | 6 | 1.7 |  |
| Sansure HPV | 372 | 136 | 36.6 | 136 | 67 | 49.3 | 29 | 7.8 | 19 | 5.1 | 6 | 1.6 |  |
| Abbreviations: WLHIV: women living with HIV; CIN: cervical intraepithelial neoplasia, grade 1 (CIN1), grade 2 or worse (CIN2+), grade 3 or worse (CIN3+); LBC: liquid-based cytology; ASCUS+: atypical squamous cells of undetermined significance and worse; LSIL+: low-grade squamous intraepithelial lesions and worse; VIA/VILI: visual inspection with acetic acid (VIA) and/or Lugol’s iodine (VILI). | | | | | | | | | | | | | |

| **Supplementary 2 Relative clinical performance of primary screening methods in WLHIV for detection of CIN2+** | | | | | | |
| --- | --- | --- | --- | --- | --- | --- |
| **Programs** | **Relative SE (95%CI)** | **Relative SP (95%CI)** | **Relative PPV (95%CI)** | **Relative NPV (95%CI)** | **Relative referral rate (95%CI)** | **Relative detection rate (95%CI)** |
| **Physician-sampling** |  |  |  |  |  |  |
| LBC (ASCUS+) | 1 | 1 | 1 | 1 | 1 | 1 |
| HC2 | 1.64 (1.21–2.80) | 0.83 (0.79–0.87) | 0.46 (0.32–0.67) | 1.02 (1.01–1.04) | 3.54 (2.56–5.26) | 1.64 (1.21–2.80) |
| cobas | 1.45 (1.12–2.42) | 0.88 (0.84–0.92) | 0.51 (0.35–0.74) | 1.01 (1.00–1.03) | 2.83 (2.09–4.15) | 1.45 (1.12–2.42) |
| Sansure HPV | 1.64 (1.21–2.80) | 0.75 (0.70–0.79) | 0.34 (0.23–0.50) | 1.02 (1.01–1.04) | 4.83 (3.42–7.28) | 1.64 (1.21–2.80) |
| VIA/VILI | 1.00 (1.00–6.32) | 0.80 (0.73–0.86) | 0.21 (0.05–0.78) | 1.00 (0.98–1.02) | 4.88 (2.74–10.76) | 1.00 (1.00–6.32) |
| LBC (LSIL+) | 1 | 1 | 1 | 1 | 1 |  |
| HC2 | 2.57 (1.56–5.78) | 0.82 (0.78–0.86) | 0.45 (0.28–0.74) | 1.03 (1.01–1.05) | 5.67 (3.65–9.78) | 2.57 (1.56–5.78) |
| cobas | 2.29 (1.43–5.06) | 0.87 (0.83–0.90) | 0.50 (0.31–0.83) | 1.03 (1.01–1.04) | 4.53 (2.96–7.75) | 2.29 (1.43–5.06) |
| Sansure HPV | 2.57 (1.56–5.78) | 0.74 (0.69–0.78) | 0.33 (0.20–0.55) | 1.03 (1.01–1.05) | 7.73 (4.89–13.47) | 2.57 (1.56–5.78) |
| VIA/VILI | 1.00 (1.00–6.32) | 0.79 (0.72–0.85) | 0.13 (0.03–0.49) | 1.00 (0.98–1.02) | 7.80 (3.65–23.27) | 1.00 (1.00–6.32) |
| HC2 | 1 | 1 | 1 | 1 | 1 |  |
| Sansure HPV | 1.00 (1.00–1.23) | 0.90 (0.86–0.93) | 0.75 (0.66–0.85) | 1.00 (NA)^*^ | 1.33 (1.21–1.51) | 1.00 (1.00–1.23) |
| cobas | 1 | 1 | 1 | 1 | 1 |  |
| Sansure HPV | 1.12 (1.01–1.50) | 0.84 (0.80–0.88) | 0.65 (0.54–0.79) | 1.01 (1.00–1.02) | 1.71 (1.48–2.04) | 1.12 (1.01–1.50) |
| **Self-sampling** |  |  |  |  |  |  |
| cobas-physician | 1 | 1 | 1 | 1 | 1 | 1 |
| cobas-self | 1.12 (1.01–1.53) | 0.85 (0.80–0.89) | 0.67 (0.54–0.82) | 1.01 (1.00–1.02) | 1.69 (1.46–2.01) | 1.12 (1.01–1.53) |
| Sansure HPV-physician | 1 | 1 | 1 | 1 | 1 |  |
| Sansure HPV-self | 1.00 (1.00–1.21) | 0.93 (0.89–0.96) | 0.87 (0.79–0.95) | 1.00 (1.00–1.00) | 1.15 (1.09–1.25) | 1.00 (1.00–1.21) |
| Abbreviations: WLHIV: women living with HIV; CIN2+: cervical intraepithelial neoplasia grade 2 or worse; SE: sensitivity; CI: confidence interval; SP: Specificity; PPV: positive predictive value; NPV: negative predictive value; LBC: liquid-based cytology; ASCUS+: atypical squamous cells of undetermined significance and worse; LSIL: low-grade squamous intraepithelial lesions and worse; VIA/VILI: visual inspection with acetic acid (VIA) and/or Lugol’s iodine (VILI); NA*: invalid for calculation. | | | | | | |

| **Supplementary 3 Clinical performance of primary screening methods in WLHIV for detection of CIN3+** | | | | | | |
| --- | --- | --- | --- | --- | --- | --- |
| **Programs** | **Sensitivity%**  **(95%CI)** | **Specificity% (95%CI)** | **PPV%**  **(95%CI)** | **NPV%**  **(95%CI)** | **AUC**  **(95%CI)** | **Referral rate% (95%CI)** |
| **Physician-sampling** |  |  |  |  |  |  |
| LBC (ASCUS+) | 100 (54.1–100) | 95.1 (92.3–97.0) | 25.0 (9.8–46.7) | 100 (98.9–100) | 0.98 (0.95–0.99) | 6.5 (4.4–9.5) |
| LBC (LSIL+) | 66.7 (22.3–95.7) | 97.0 (94.7–98.5) | 26.7 (7.8–55.1) | 99.4 (98–99.9) | 0.82 (0.78–0.86) | 4.1 (2.5–6.6) |
| HC2 | 100 (54.1–100) | 77.6 (72.9–81.9) | 7.1 (2.6–14.7) | 100 (98.7–100) | 0.89 (0.85–0.92) | 23.7 (19.6–28.3) |
| cobas | 100 (54.1–100) | 82.6 (78.3–86.4) | 8.7 (3.3–18) | 100 (98.8–100) | 0.91 (0.88–0.94) | 18.7 (15.1–23.0) |
| Sansure HPV | 100 (54.1–100) | 69.4 (64.4–74.1) | 5.1 (1.9–10.7) | 100 (98.6–100) | 0.85 (0.81–0.88) | 31.7 (27.2–36.6) |
| **Self-sampling** |  |  |  |  |  |  |
| cobas | 100 (54.1–100) | 69.5 (64.4–74.2) | 5.3 (2–11.1) | 100 (98.5–100) | 0.85 (0.81–0.88) | 31.7 (27.1–36.6) |
| Sansure HPV | 100 (54.1–100) | 64.5 (59.3–69.4) | 4.4 (1.6–9.4) | 100 (98.4–100) | 0.82 (0.78–0.86) | 36.6 (31.8–41.6) |
| Abbreviations: WLHIV: women living with HIV; CIN3+: cervical intraepithelial neoplasia grade 3 or worse; CI: confidence interval; PPV: positive predictive value; NPV: negative predictive value; AUC: area under the curve; LBC: liquid-based cytology; ASCUS+: atypical squamous cells of undetermined significance or worse; LSIL: low-grade squamous intraepithelial lesions or worse; VIA/VILI: visual inspection with acetic acid (VIA) or Lugol’s iodine (VILI). | | | | | | |

| **Supplementary 4 Relative clinical performance of primary screening methods in WLHIV for detection of CIN3+** | | | | | | |
| --- | --- | --- | --- | --- | --- | --- |
| **Programs** | **Relative SE (95%CI)** | **Relative SP (95%CI)** | **Relative PPV (95%CI)** | **Relative NPV (95%CI)** | **Relative referral rate (95%CI)** | **Relative detection rate (95%CI)** |
| **Physician-sampling** |  |  |  |  |  |  |
| LBC (ASCUS+) | 1 | 1 | 1 | 1 | 1 | 1 |
| HC2 | 1.00 (1.00–1.85) | 0.82 (0.77–0.86) | 0.28 (0.20–0.40) | 1.00 (NA)^*^ | 3.54 (2.56–5.26) | 1.00 (1.00–1.85) |
| cobas | 1.00 (1.00–1.85) | 0.87 (0.83–0.91) | 0.35 (0.25–0.50) | 1.00 (1.00–1.00) | 2.83 (2.09–4.15) | 1.00 (1.00–1.85) |
| Sansure HPV | 1.00 (1.00–1.85) | 0.73 (0.68–0.78) | 0.21 (0.14–0.30) | 1.00 (1.00–1.00) | 4.83 (3.42–7.28) | 1.00 (1.00–1.85) |
| LBC (LSIL+) | 1 | 1 | 1 | 1 | 1 | 1 |
| HC2 | 1.50 (1.05–4.49) | 0.80 (0.75–0.84) | 0.26 (0.14–0.49) | 1.01 (1.00–1.01) | 5.67 (3.65–9.78) | 1.50 (1.05–4.49) |
| cobas | 1.50 (1.05–4.49) | 0.85 (0.81–0.89) | 0.33 (0.18–0.61) | 1.01 (1.00–1.01) | 4.53 (2.96–7.75) | 1.50 (1.05–4.49) |
| Sansure HPV | 1.50 (1.05–4.49) | 0.72 (0.67–0.77) | 0.19 (0.10–0.36) | 1.01 (1.00–1.01) | 7.73 (4.89–13.47) | 1.50 (1.05–4.49) |
| HC2 | 1 | 1 | 1 | 1 | 1 | 1 |
| Sansure HPV | 1.00 (1.00–1.85) | 0.90 (0.86–0.93) | 0.75 (0.66–0.85) | 1.00 (NA)^*^ | 1.33 (1.21–1.51) | 1.00 (1.00–1.85) |
| cobas | 1 | 1 | 1 | 1 | 1 | 1 |
| Sansure HPV | 1.00 (1.00–1.85) | 0.84 (0.79–0.88) | 0.58 (0.50–0.69) | 1.00 (1.00–1.00) | 1.71 (1.48–2.04) | 1.00 (1.00–1.85) |
| **Self-sampling** |  |  |  |  |  |  |
| cobas-physician | 1 | 1 | 1 | 1 | 1 | 1 |
| cobas-self | 1.00 (1.00–1.85) | 0.84 (0.79–0.88) | 0.59 (0.50–0.70) | 1.00 (1.00–1.00) | 1.69 (1.46–2.01) | 1.00 (1.00–1.85) |
| Sansure HPV-physician | 1 | 1 | 1 | 1 | 1 | 1 |
| Sansure HPV-self | 1.00 (1.00–1.85) | 0.93 (0.89–0.96) | 0.87 (0.79–0.95) | 1.00 (1.00–1.00) | 1.15 (1.09–1.25) | 1.00 (1.00–1.85) |
| Abbreviations: WLHIV: women living with HIV; CIN3+: cervical intraepithelial neoplasia grade 3 or worse; SE: sensitivity; CI: confidence interval; SP: Specificity; PPV: positive predictive value; NPV: negative predictive value; LBC: liquid-based cytology; ASCUS+: atypical squamous cells of undetermined significance and worse; LSIL: low-grade squamous intraepithelial lesions and worse; VIA/VILI: visual inspection with acetic acid (VIA) and/or Lugol’s iodine (VILI); NA*: invalid for calculation. | | | | | | |

| **Supplementary 5 Basic information for triage strategies in WLHIV** | | | | | | | | | | | | |
| --- | --- | --- | --- | --- | --- | --- | --- | --- | --- | --- | --- | --- |
| **Strategies** | **No. screened** | **No.**  **screen positive** | **%** | **No. with colposcopy** | **No. with**  **abnormal colposcopy** | **%** | **No. CIN1** | **%** | **No. CIN2+** | % | **No. CIN3+** | % |
| **Physician-sampling** |  |  |  |  |  |  |  |  |  |  |  |  |
| HC2 |  |  |  |  |  |  |  |  |  |  |  |  |
| LBC (ASCUS+) | 359 | 20 | 5.6 | 20 | 14 | 70.0 | 4 | 4.7 | 11 | 3.1 | 6 | 7.1 |
| LBC (LSIL+) | 359 | 13 | 3.6 | 13 | 8 | 61.5 | 2 | 2.4 | 7 | 1.9 | 4 | 4.7 |
| VIA/VILI | 168 | 10 | 6.0 | 10 | 6 | 60.0 | 3 | 8.8 | 2 | 1.2 | 0 | 0 |
| cobas |  |  |  |  |  |  |  |  |  |  |  |  |
| HPV-16/18 | 369 | 26 | 7.1 | 26 | 20 | 76.9 | 2 | 2.9 | 13 | 3.5 | 6 | 8.7 |
| LBC (ASCUS+) | 369 | 19 | 5.2 | 19 | 14 | 73.7 | 4 | 5.8 | 11 | 3.0 | 6 | 8.7 |
| LBC (LSIL+) | 369 | 12 | 3.3 | 12 | 8 | 66.7 | 2 | 2.9 | 7 | 1.9 | 4 | 5.8 |
| HPV-16/18\|LBC (ASCUS+) | 369 | 34 | 9.2 | 34 | 25 | 73.5 | 6 | 8.7 | 15 | 4.1 | 6 | 8.7 |
| VIA/VILI | 168 | 8 | 4.8 | 8 | 6 | 75.0 | 3 | 14.3 | 2 | 1.2 | 0 | 0 |
| Sansure HPV |  |  |  |  |  |  |  |  |  |  |  |  |
| HPV-16/18 | 372 | 31 | 8.3 | 31 | 22 | 71.0 | 3 | 2.5 | 13 | 3.5 | 6 | 5.1 |
| HPV-16/18/31/33/45/52/58 | 372 | 81 | 21.8 | 81 | 51 | 63.0 | 18 | 15.3 | 17 | 4.6 | 6 | 5.1 |
| LBC (ASCUS+) | 372 | 20 | 5.4 | 20 | 14 | 70.0 | 4 | 3.4 | 11 | 3.0 | 6 | 5.1 |
| LBC (LSIL+) | 372 | 13 | 3.5 | 13 | 8 | 61.5 | 2 | 1.7 | 7 | 1.9 | 4 | 3.4 |
| HPV-16/18\|LBC (ASCUS+) | 372 | 40 | 10.8 | 40 | 26 | 65.0 | 7 | 5.9 | 15 | 4.0 | 6 | 5.1 |
| VIA/VILI | 168 | 11 | 6.6 | 11 | 7 | 63.6 | 3 | 5.9 | 2 | 1.2 | 0 | 0 |
| **Self-sampling** |  |  |  |  |  |  |  |  |  |  |  |  |
| cobas |  |  |  |  |  |  |  |  |  |  |  |  |
| HPV-16/18 | 360 | 39 | 10.8 | 39 | 26 | 66.7 | 8 | 7.0 | 13 | 3.6 | 6 | 5.3 |
| Sansure HPV |  |  |  |  |  |  |  |  |  |  |  |  |
| HPV-16/18 | 372 | 34 | 9.1 | 34 | 21 | 61.8 | 3 | 2.2 | 14 | 3.8 | 6 | 4.4 |
| HPV-16/18/31/33/45/52/58 | 372 | 98 | 26.3 | 98 | 53 | 54.1 | 22 | 16.2 | 18 | 4.8 | 6 | 4.4 |
| Abbreviations: WLHIV: women living with HIV; CIN: cervical intraepithelial neoplasia, grade 1 (CIN1), grade 2 and worse (CIN2+), grade 3 and worse (CIN3+); LBC: liquid cytology; ASCUS+: atypical squamous cells of undetermined significance and worse; LSIL: low-grade squamous intraepithelial lesions and worse; VIA/VILI: visual inspection with acetic acid (VIA) and/or Lugol’s iodine (VILI) | | | | | | | | | | | | |

| **Supplementary 6 Relative clinical performance of triage strategies in HPV-positive WLHIV for detection of CIN2+** | | | | | | |
| --- | --- | --- | --- | --- | --- | --- |
| **Programs** | **Relative SE (95%CI)** | **Relative SP (95%CI)** | **Relative PPV (95%CI)** | **Relative NPV (95%CI)** | **Relative referral rate (95%CI)** | **Relative detection rate (95%CI)** |
| **Physician-sampling** |  |  |  |  |  |  |
| HC2 without triage | 1 | 1 | 1 | 1 | 1 | 1 |
| LBC (ASCUS+) | 0.61 (0.36–0.83) | 1.21 (1.16–1.28) | 2.60 (1.80–3.74) | 0.98 (0.96–0.99) | 0.24 (0.15–0.34) | 0.61 (0.36–0.83) |
| LBC (LSIL+) | 0.39 (0.17–0.64) | 1.22 (1.17–1.29) | 2.54 (1.58–4.08) | 0.97 (0.95–0.99) | 0.15 (0.08–0.25) | 0.39 (0.17–0.64) |
| VIA/VILI | 0.40 (0.05–0.85) | 1.17 (1.10–1.27) | 1.36 (0.51–3.63) | 0.98 (0.96–1.00) | 0.29 (0.15–0.47) | 0.40 (0.05–0.85) |
| cobas without triage | 1 | 1 | 1 | 1 | 1 | 1 |
| HPV-16/18 | 0.76 (0.50–0.93) | 1.13 (1.09–1.18) | 2.03 (1.50–2.75) | 0.99 (0.98–1.00) | 0.38 (0.26–0.50) | 0.76 (0.50–0.93) |
| LBC (ASCUS+) | 0.65 (0.38–0.86) | 1.15 (1.11–1.20) | 2.35 (1.65–3.36) | 0.98 (0.97–1.00) | 0.28 (0.17–0.40) | 0.65 (0.38–0.86) |
| LBC (LSIL+) | 0.41 (0.18–0.67) | 1.16 (1.11–1.22) | 2.37 (1.50–3.75) | 0.97 (0.96–0.99) | 0.17 (0.09–0.28) | 0.41 (0.18–0.67) |
| HPV-16/18\|LBC (ASCUS+) | 0.88 (0.64–0.99) | 1.11 (1.07–1.16) | 1.79 (1.40–2.29) | 0.99 (0.99–1.00) | 0.49 (0.37–0.62) | 0.88 (0.64–0.99) |
| VIA/VILI | 0.50 (0.07–0.93) | 1.08 (1.04–1.14) | 1.31 (0.54–3.17) | 0.99 (0.97–1.01) | 0.38 (0.18–0.62) | 0.40 (0.05–0.85) |
| Sansure HPV without triage | 1 | 1 | 1 | 1 | 1 | 1 |
| HPV-16/18 | 0.68 (0.43–0.87) | 1.32 (1.25–1.41) | 2.60 (1.88–3.61) | 0.98 (0.97–1.00) | 0.26 (0.19–0.35) | 0.68 (0.43–0.87) |
| HPV-16/18/31/33/45/52/58 | 0.89 (0.67–0.99) | 1.14 (1.09–1.20) | 1.30 (1.10–1.54) | 0.99 (0.98–1.00) | 0.69 (0.59–0.77) | 0.89 (0.67–0.99) |
| LBC (ASCUS+) | 0.58 (0.33–0.80) | 1.35 (1.28–1.45) | 3.42 (2.33–5.00) | 0.98 (0.96–0.99) | 0.17 (0.11–0.25) | 0.58 (0.33–0.80) |
| LBC (LSIL+) | 0.37 (0.16–0.62) | 1.37 (1.29–1.47) | 3.34 (2.06–5.44) | 0.97 (0.95–0.99) | 0.11 (0.06–0.18) | 0.37 (0.16–0.62) |
| HPV-16/18\|LBC (ASCUS+) | 0.79 (0.54–0.94) | 1.29 (1.22–1.38) | 2.33 (1.76–3.07) | 0.99 (0.98–1.00) | 0.34 (0.25–0.43) | 0.79 (0.54–0.94) |
| VIA/VILI | 0.40 (0.05–0.85) | 1.32 (1.21–1.46) | 1.85 (0.68–5.06) | 0.98 (0.96–1.00) | 0.22 (0.11–0.35) | 0.40 (0.05–0.85) |
| **Self-sampling** |  |  |  |  |  |  |
| cobas without triage | 1 | 1 | 1 | 1 | 1 | 1 |
| HPV-16/18 | 0.72 (0.47–0.90) | 1.28 (1.21–1.37) | 2.11 (1.56–2.86) | 0.98 (0.97–1.00) | 0.34 (0.26–0.44) | 0.72 (0.47–0.90) |
| Sansure HPV without triage | 1 | 1 | 1 | 1 | 1 |  |
| HPV-16/18 | 0.74 (0.49–0.91) | 1.41 (1.32–1.52) | 2.95 (2.16–4.03) | 0.99 (0.97–1.00) | 0.25 (0.18–0.33) | 0.74 (0.49–0.91) |
| HPV-16/18/31/33/45/52/58 | 0.95 (0.74–1.00) | 1.16 (1.11–1.22) | 1.31 (1.15–1.50) | 1.00 (0.99–1.00) | 0.72 (0.64–0.79) | 0.95 (0.74–1.00) |
| Abbreviations: WLHIV: women living with HIV; CIN2+: cervical intraepithelial neoplasia grade 2 or worse; SE: sensitivity; CI: confidence interval; SP: Specificity; PPV: positive predictive value; NPV: negative predictive value; LBC: liquid-based cytology; ASCUS+: atypical squamous cells of undetermined significance and worse; LSIL+: low-grade squamous intraepithelial lesions and worse; VIA/VILI: visual inspection with acetic acid (VIA) and/or Lugol’s iodine (VILI). | | | | | | |

| **Supplementary 7: Clinical performance of triage strategies in HPV-positive WLHIV for detection of CIN3+** | | | | | | |
| --- | --- | --- | --- | --- | --- | --- |
| **Programs** | **Sensitivity% (95%CI)** | **Specificity% (95%CI)** | **PPV%**  **(95%CI)** | **NPV%**  **(95%CI)** | **AUC%**  **(95%CI)** | **Referral rate% (95%CI)** |
| **Physician-sampling** |  |  |  |  |  |  |
| For HC2-positive |  |  |  |  |  |  |
| LBC (ASCUS+) | 100 (54.1–100) | 96.0 (93.4–97.8) | 30.0 (11.9–54.3) | 100 (98.9–100) | 0.98 (0.96–0.99) | 5.6 (3.6–8.5) |
| LBC (LSIL+) | 66.7 (22.3–95.7) | 97.5 (95.2–98.8) | 30.8 (9.1–61.4) | 99.4 (97.9–99.9) | 0.82 (0.78–0.86) | 3.6 (2.1–6.1) |
| For cobas-positive |  |  |  |  |  |  |
| HPV-16/18 | 100 (54.1–100) | 94.5 (91.6–96.6) | 23.1 (9.0–43.6) | 100 (98.9–100) | 0.97 (0.95–0.99) | 7.0 (4.9–10.1) |
| LBC (ASCUS+) | 100 (54.1–100) | 96.4 (94.0–98.1) | 31.6 (12.6–56.6) | 100 (99.0–100) | 0.98 (0.96–0.99) | 5.1 (3.3–7.9) |
| LBC (LSIL+) | 66.7 (22.3–95.7) | 97.8 (95.7–99.0) | 33.3 (9.9–65.1) | 99.4 (98.0–99.9) | 0.82 (0.78–0.86) | 3.3 (1.9–5.6) |
| HPV-16/18\|LBC (ASCUS+) | 100 (54.1–100) | 92.3 (89–94.8) | 17.6 (6.8–34.5) | 100 (98.9–100) | 0.96 (0.94–0.98) | 9.2 (6.7–12.6) |
| For Sansure HPV-positive |  |  |  |  |  |  |
| HPV-16/18 | 100 (54.1–100) | 93.2 (90.1–95.5) | 19.4 (7.5–37.5) | 100 (98.9–100) | 0.97 (0.94–0.98) | 8.3 (5.9–11.6) |
| HPV-16/18/31/33/45/52/58 | 100 (54.1–100) | 79.5 (75.0–83.5) | 7.4 (2.8–15.4) | 100 (98.7–100) | 0.90 (0.86–0.93) | 21.8 (17.9–26.2) |
| LBC (ASCUS+) | 100 (54.1–100) | 96.2 (93.7–97.9) | 30.0 (11.9–54.3) | 100 (99.0–100) | 0.98 (0.96–0.99) | 5.4 (3.5–8.2) |
| LBC (LSIL+) | 66.7 (22.3–95.7) | 97.5 (95.4–98.9) | 30.8 (9.1–61.4) | 99.4 (98.0–99.9) | 0.82 (0.78–0.86) | 3.5 (2.1–5.9) |
| HPV-16/18\|LBC (ASCUS+) | 100 (54.1–100) | 90.7 (87.3–93.5) | 15.0 (5.7–29.8) | 100 (98.9–100) | 0.95 (0.93–0.97) | 10.8 (8.0–14.3) |
| **Self-sampling** |  |  |  |  |  |  |
| For cobas-positive |  |  |  |  |  |  |
| HPV-16/18 | 100 (54.1–100) | 90.7 (87.2–93.5) | 15.4 (5.9–30.5) | 100 (98.9–100) | 0.95 (0.93–0.97) | 10.8 (8.0–14.5) |
| For Sansure HPV-positive |  |  |  |  |  |  |
| HPV-16/18 | 100 (54.1–100) | 92.3 (89.1–94.9) | 17.6 (6.8–34.5) | 100 (98.9–100) | 0.96 (0.94–0.98) | 9.1 (6.6–12.5) |
| HPV-16/18/31/33/45/52/58 | 100 (54.1–100) | 74.9 (70.1–79.2) | 6.1 (2.3–12.9) | 100 (98.7–100) | 0.87 (0.84–0.91) | 26.3 (22.1–31.0) |
| Abbreviations: WLHIV: women living with HIV; CIN3+: cervical intraepithelial neoplasia grade 3 or worse; CI: confidence interval; PPV: positive predictive value; NPV: negative predictive value; AUC: area under the curve; LBC: liquid-based cytology; ASCUS+: atypical squamous cells of undetermined significance or worse; LSIL: low-grade squamous intraepithelial lesions or worse; VIA/VILI: visual inspection with acetic acid (VIA) or Lugol’s iodine (VILI). | | | | | | |

| **Supplementary 8 Relative clinical performance of triage strategies in HPV-positive WLHIV for detection of CIN3+** | | | | | | |
| --- | --- | --- | --- | --- | --- | --- |
| **Programs** | **Relative SE (95%CI)** | **Relative SP (95%CI)** | **Relative PPV (95%CI)** | **Relative NPV (95%CI)** | **Relative referral rate (95%CI)** | **Relative detection rate (95%CI)** |
| **Physician-sampling** |  |  |  |  |  |  |
| HC2 without triage | 1 | 1 | 1 | 1 | 1 | 1 |
| LBC (ASCUS+) | 1.00 (1.00–1.85) | 1.24 (1.18–1.31) | 4.25 (2.90–6.23) | 1.00 (1.00–1.00) | 0.24 (0.15–0.34) | 1.00 (1.00–1.85) |
| LBC (LSIL+) | 0.67 (0.22–0.96) | 1.26 (1.19–1.33) | 4.36 (2.37–8.03) | 0.99 (0.99–1.00) | 0.15 (0.08–0.25) | 0.67 (0.22–0.96) |
| cobas without triage | 1 | 1 | 1 | 1 | 1 | 1 |
| HPV-16/18 | 1.00 (1.00–1.85) | 1.14 (1.10–1.20) | 2.65 (1.96–3.59) | 1.00 (1.00–1.00) | 0.38 (0.26–0.50) | 1.00 (1.00–1.85) |
| LBC (ASCUS+) | 1.00 (1.00–1.85) | 1.17 (1.12–1.23) | 3.63 (2.48–5.33) | 1.00 (1.00–1.00) | 0.28 (0.17–0.40) | 1.00 (1.00–1.85) |
| LBC (LSIL+) | 0.67 (0.22–0.96) | 1.18 (1.13–1.25) | 3.83 (2.08–7.05) | 0.99 (0.99–1.00) | 0.17 (0.09–0.28) | 0.67 (0.22–0.96) |
| HPV-16/18\|LBC (ASCUS+) | 1.00 (1.00–1.85) | 1.12 (1.08–1.17) | 2.03 (1.60–2.58) | 1.00 (1.00–1.00) | 0.49 (0.37–0.62) | 1.00 (1.00–1.85) |
| Sansure HPV without triage | 1 | 1 | 1 | 1 | 1 | 1 |
| HPV-16/18 | 1.00 (1.00–1.85) | 1.34 (1.27–1.44) | 3.81 (2.81–5.15) | 1.00 (1.00–1.00) | 0.26 (0.19–0.35) | 1.00 (1.00–1.85) |
| HPV-16/18/31/33/45/52/58 | 1.00 (1.00–1.85) | 1.15 (1.10–1.21) | 1.46 (1.29–1.65) | 1.00 (1.00–1.00) | 0.69 (0.59–0.77) | 1.00 (1.00–1.85) |
| LBC (ASCUS+) | 1.00 (1.00–1.85) | 1.39 (1.30–1.49) | 5.90 (3.96–8.80) | 1.00 (1.00–1.00) | 0.17 (0.11–0.25) | 1.00 (1.00–1.85) |
| LBC (LSIL+) | 0.67 (0.22–0.96) | 1.41 (1.32–1.51) | 6.05 (3.25–11.26) | 0.99 (0.99–1.00) | 0.11 (0.06–0.18) | 0.67 (0.22–0.96) |
| HPV-16/18\|LBC (ASCUS+) | 1.00 (1.00–1.85) | 1.31 (1.24–1.40) | 2.95 (2.29–3.80) | 1.00 (1.00–1.00) | 0.34 (0.25–0.43) | 1.00 (1.00–1.85) |
| **Self-sampling** |  |  |  |  |  |  |
| cobas without triage | 1 | 1 | 1 | 1 | 1 | 1 |
| HPV-16/18 | 1.00 (1.00–1.85) | 1.30 (1.23–1.40) | 2.92 (2.27–3.77) | 1.00 (1.00–1.00) | 0.34 (0.26–0.44) | 1.00 (1.00–1.85) |
| Sansure HPV without triage | 1 | 1 | 1 | 1 | 1 | 1 |
| HPV-16/18 | 1.00 (1.00–1.85) | 1.43 (1.34–1.55) | 4.00 (2.99–5.35) | 1.00 (1.00–1.00) | 0.25 (0.18–0.33) | 1.00 (1.00–1.85) |
| HPV-16/18/31/33/45/52/58 | 1.00 (1.00–1.85) | 1.16 (1.11–1.23) | 1.39 (1.25–1.54) | 1.00 (1.00–1.00) | 0.72 (0.64–0.79) | 1.00 (1.00–1.85) |
| Abbreviations: WLHIV: women living with HIV; CIN3+: cervical intraepithelial neoplasia grade 3 or worse; SE: sensitivity; CI: confidence interval; SP: Specificity; PPV: positive predictive value; NPV: negative predictive value; LBC: liquid-based cytology; ASCUS+: atypical squamous cells of undetermined significance and worse; LSIL+: low-grade squamous intraepithelial lesions and worse; VIA/VILI: visual inspection with acetic acid (VIA) and/or Lugol’s iodine (VILI). | | | | | | |

| **Supplementary 9 Univariate and multivariate logistic regression analysis for specificity of screening methods in WLHIV** | | | | | | | | | | | |  |
| --- | --- | --- | --- | --- | --- | --- | --- | --- | --- | --- | --- | --- |
| **Strata** | **LBC (ASCUS+)** | |  | **HC2** | |  | **cobas** | |  | **Sansure HPV** | | |
|  | **cOR (95%CI)** | **aOR (95%CI)** |  | **cOR (95%CI)** | **aOR (95%CI)** |  | **cOR (95%CI)** | **aOR (95%CI)** |  | **cOR (95%CI)** | **aOR (95%CI)** | |
| **CD4 count** |  |  |  |  |  |  |  |  |  |  |  | |
| ≥350 cell/µL | 1 | 1 |  | 1 | 1 |  | 1 | 1 |  | 1 | 1 | |
| <350 cell/µL | **2.51**  **(1.02-6.22)** | 2.64  (0.98-7.15) |  | **2.81**  **(1.53-5.15)** | 1.88  (0.94-3.77) |  | 1.86  (0.96-3.58) | 1.28  (0.60-2.75) |  | **2.03**  **(1.15-3.57)** | 1.26  (0.66-2.42) | |
| **cART duration** |  |  |  |  |  |  |  |  |  |  |  | |
| >2 years | 1 | 1 |  | 1 | 1 |  | 1 | 1 |  | 1 | 1 | |
| ≤2 years | 1.36  (0.56-3.30) | 1.21  (0.46-3.21) |  | **2.69**  **(1.56-4.63)** | **1.87**  **(1.01-3.46)** |  | **2.18**  **(1.22-3.91)** | 1.75  (0.90-3.40) |  | **2.92**  **(1.78-4.80)** | **2.48**  **(1.43-4.29)** | |
| **Age** |  |  |  |  |  |  |  |  |  |  |  | |
| ≥40 years | 1 | 1 |  | 1 | 1 |  | 1 | 1 |  | 1 | 1 | |
| <40 years | 2.12  (0.91-4.99) | 1.85  (0.75-4.57) |  | 0.68  (0.47-1.12) | 0.71  (0.40-1.27) |  | 0.75  (0.44-1.27) | 0.76  (0.40-1.41) |  | **0.62**  **(0.40-0.98)** | 0.69  (0.41-1.16) | |
| Abbreviations: WLHIV: women living with HIV; LBC: liquid-based cytology; ASCUS+: atypical squamous cells of undetermined significance or worse; cOR: crude odds ratio; CI: confidence interval; aOR: adjusted odds ratio; cART: combination antiretroviral therapy. | | | | | | | | | | | |  |
